# Patterns of common skin infections among children living with HIV/AIDS in Hawassa City, Ethiopia: a cross sectional study

**DOI:** 10.1186/s13104-018-3991-4

**Published:** 2018-12-12

**Authors:** Bereket Duko, Melese Gebrie, Bedilu Deribe, Asres Bedaso, Mohammed Ayalew

**Affiliations:** Faculty of Health Sciences, College of Medicine and Health Sciences, Hawassa University, Hawassa, Ethiopia

**Keywords:** Pattern, Skin infection, Children, HIV/AIDS, Ethiopia

## Abstract

**Objectives:**

Skin disorders are the most common health problems seen among HIV positive patients. It presents with a variety of manifestations which can cause significant morbidity. This study was aimed to assess the prevalence of common skin problems among children living with HIV/AIDS at Hawassa University Comprehensive Specialized Hospital, Hawassa, Ethiopia, 2017/2018. Hospital based cross-sectional study was conducted among 125 children living with HIV/AIDS who were recruited through simple random sampling techniques from February to April 2017. Pre-tested, structured questionnaires were used to collect the data.

**Result:**

Among a total of 125 study participants, 72 (57.6%) of the children were males and 97 (77.6%) were in the age range of 10–14 years. 90 (72%) of participants had different kinds of skin problems. Among those who had one kind of common skin infection, 53 (42.4%) were males. Viral skin infections that accounts 48 (53.3%), were the leading cause of skin infections followed by 43 (47.8%), 33(36.7%) and 22 (24.7%) fungal infections, inflammatory and bacterial skin infections respectively. Among all children who were taking ART, only 2.4% of the children had skin related side effects.

**Electronic supplementary material:**

The online version of this article (10.1186/s13104-018-3991-4) contains supplementary material, which is available to authorized users.

## Introduction

HIV/AIDS is the most common and serious viral disease caused by human immunodeficiency virus (HIV) that affects about 39–46 million people in the world [1, 2]. Most reside in the developing world, with approximately two-third in sub-Saharan Africa and nearly 12% of these infections occurred in children younger than 15 years of age [3]. The USA and Europe have documented transmission rates in untreated women between 12 and 30%. Transmission rates in Africa are higher that ranges from 25 to 52% [4].

Skin infections account the most common clinical manifestations in children. About 90% of patients develop at least one type of skin disease during the course of their illness and more than one-third of patients present with skin lesions as a marker of HIV infection [5]. Skin and mucocutaneous infections such as herpes simplex infection, candidiasis, impetigo, ecthyma and furun-cles, mollus-cum contagiosum, plane warts, seborrheic eczema and Kaposi’s sarco-ma etc. are common skin infections among people living with HIV/AIDS [2]. Skin disorders in children living with HIV/AIDS may have atypical presentation, be inclined to be more severe and may diagnosed wrongly [6].

Children living with HIV can develop different infectious and inflammatory diseases of the skin. These skin or mucocutaneous disorders may provide an early clue to the presence of pediatric HIV infection and often more severe and more difficult to treat than in the immune-competent child. Skin disorders are common in children in Ethiopia, where nearly four out of five (72.6–79%) children living with HIV/AIDS develop at least one skin type infection [6, 7].

Immune reconstruction induced by anti-retroviral therapy (ART) would be anticipated to decrease the prevalence of many opportunistic infections including skin disorders due to inhibition of viral replication [8]. Hence, skin infections are major health problems among children living with HIV presenting with a variety of dermatologic man-ifestations. In this study, our aim was to as-sess pattern of skin infections among children living with HIV/AIDS.

## Main text

### Study design and setting

This descriptive cross-sectional study was conducted among children living with HIV/AIDS at Hawassa University comprehensive specialized hospital, ART clinic from February to April 2017. The sample size was calculated using a single population proportion formula was used to calculate required sample size. A total of 270 children were registered in ART registry and actively taking ART drugs. Among those children who fulfilled the inclusion criteria, total of 125 children living with HIV/AIDS were recruited using simple random sampling techniques by computer generated number using the ART registry number as sampling frame.

### Data collection

Interviewer administered structured and pre-tested questionnaires were used to collect the data. The questionnaire had four parts such as socio demographic data of the child, socio demographic data of the care giver, ART Treatment related factors and skin problems in children related questions. A standardized clinical history was documented and all participants undertake a comprehensive dermatologic examination by the dermatologists and expert nurse clinicians, as part of a full clinical evaluation including WHO staging. Clinical dermatological evaluation was done in daylight and majority of the diagnoses of skin infections were done clinically. Laboratory tests that are appropriate to diagnose skin infections like KOH, gram stain, culture and sensitivity were done to affirm the diagnosis, when necessary.

### Data analysis

Data were checked for completeness and consistency, then entered and cleaned using Epi-Data version 3.0. Statistical analysis was done using the Statistical Package for Social Sciences (SPSS) program version 20. Results were expressed as means and standard deviations and frequencies and percentages.

### Result

#### Socio-demographic characteristics of the children

Among a total of 125 study participants, 72 (57.6%) of the children were males and 97 (77.6%) were in the age between 10 and 14 years. 91 (75.8%) of the children’s were attending primary education (grade 1–8) and majority 88 (70.4%) of the children were fully immunized their vaccination (Table 1).

**Table 1:** Socio-demographic characteristics of the children living with HIV/AIDS in Hawassa Comprehensive Specialized Hospital, Hawassa, Ethiopia, 2018 (N = 125)

Variables	Categories	Number	Percent (%)
Age	0–4 years	4	3.2
	5–9 years	24	19.2
	10–14 years	97	77.6
Sex	Male	72	57.6
	Female	53	42.4
Level of education (N = 121)	Kindergarten education	4	3.3
	Primary education	91	75.2
	Secondary education	26	21.5
Immunization status	Fully immunized	88	70.4
	Immunized some	33	26.4
	Non-immunized	4	3.2

#### Socio-demographic characteristics of the care takers

Out of 125 caretakers of children, 96 (76.8%) were female, 65 (52%) were in age group 25–34 years, 114 (91.2%) were from urban residence, 42 (33.6%) and 38 (30.4%) of caretakers were Amhara and Oromo by ethnicity, respectively (Table 2).

**Table 2:** Socio-demographic characteristics of care taker of children living with HIV/AIDS in Hawassa University Comprehensive Specialized Hospital, Hawassa, Ethiopia, 2018 (N = 125)

Variables	Categories	Number	Percent (%)
Age	15–24 years	13	10.4
	25–34 years	65	52.0
	35–44 years	34	27.2
	≥ 45 years	13	10.4
Sex	Male	29	23.2
	Female	96	76.8
Residence	Urban	114	91.2
	Rural	11	8.8
Marital status	Single	10	8.0
	Married	104	83.2
	Divorced	5	4.0
	Widowed	6	4.8
Religion	Orthodox	60	48.0
	Muslim	19	15.2
	Protestant	43	34.4
	Catholic	3	2.4
Ethnicity	Amhara	42	33.6
	Oromo	38	30.4
	Sidama	22	17.6
	Wolayita	17	13.6
	Others	6	4.8
Family size	2–4	88	70.4
	5–7	33	26.4
	≥ 8	4	3.2
Level of education	Illiterate	11	8.8
	Read and write	27	21.6
	Primary education completed	37	29.6
	Secondary education completed	29	23.2
	College/University completed	21	16.8
Occupation	Merchant	22	17.6
	Gov’t employee	28	22.4
	Self-employee	39	31.2
	Daily laborer	20	17.6
	Farmer	6	4.8
	Student	5	4.0
	Other	5	4.0
Average monthly income	> 1500 EBR	32	25.6
	700–1499 EBR	60	48.0
	< 700 EBR	27	21.6
	Non specified	6	4.8
Family history of skin infection	Yes	19	15.2
	No	106	84.8

#### Prevalence of common skin infections

**Fig. 1:**
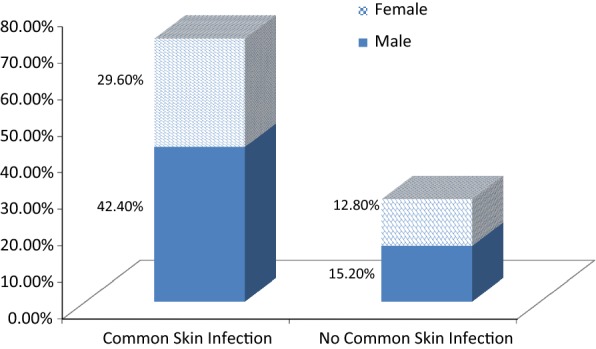
Prevalence of common skin infection with sex category of children’s living with HIV/AIDS at Hawassa University Comprehensive Specialized Hospital, Hawassa, Ethiopia, 2018

Out of all study participants, 90 (72%) had different kinds of skin problems. Of those patients who had at least one kinds of common skin infection, where 53 (42.4%) were males and 37 (29.6%) were females (Fig. 1). Among all study participants, majority 107 (85.6%) of the study participants were currently on WHO clinical stage I and, 114 (91.2%) of the children’s had been taken ART drugs for greater than or equal to 6 months. 70 (56%) of the study subjects were taken first line ART drug. Regarding drug adherence, 115 (92.0%) and 113 (90.4%) of the study participants had drug dose and schedule adherence respectively. Among all children who were taking ART, only 2.4% of the children had skin related side effects. Viral skin infections which accounts 48 (53.3%) were the leading cause of skin infections followed by 43 (47.8%), 33 (36.7%) and 22 (24.7%) fungal infections, inflammatory and bacterial skin infections, respectively (See Additional files 1, 2 and 3).

## Discussion

The aim of this study was to assess the pattern of skin problems among children living with HIV/AIDS attending in Hawassa University comprehensive specialized hospital, Hawassa, Ethiopia. Thus, skin and mucocutaneous infections can be the early indication of HIV-associated compromised immune system [2, 9]. Therefore, identification of these HIV-related skin problems may possibly lead to early diagnosis of HIV infection and endowing with timely initiation of appropri-ate ART [2].

This study showed that 72% of children had at least one type of skin disorders. Similar to our study, high prevalence of skin and mucocutaneous infections has been reported in Addis Ababa, 72.6% and 79% [6, 7], in Cameroon (68.8%) [10], in Nigeria (72%) [11], and India (67.06%) [9]. Nevertheless, the finding lower than studies from Tanzania (85%) [12], Zimbabwe 88% [13] and, India 93.7% [14] and (88.3%) [15] and higher than a study conducted in Guinea (54.62%) [16]. The variations might be due to the occurrence and pattern of skin infections vary from region to region since specific skin manifestation are common in certain regions of the world [17]. In addition, differences in climatic and environmental circumstances [14], self-care and sanitation, and variation in sample size in the different studies may affect the varied results observed.

Viral skin infections which accounts for 53.3% were the leading cause of skin infections followed by fungal infections which accounts for 47.8% and inflammatory related skin infections (36.7%). This finding is in line with a study from India where viral, fungal and bacterial skin infections took the highest prevalence [18]. The higher prevalence of these infectious dermatosis is due to the weakening of the Langerhan’s cells responsible for the mucocutaneous immunological system [19]. There has been a remarkable reduction in the opportunistic infections such as skin infections like oral candidiasis and seborrheic dermatosis with the introduction of Highly Activated Antiretroviral Therapy (HAART) [20]. However, some skin problems have paradoxically exacerbated after beginning of HAART like herpes zoster, mycobacterium infections and drug reactions [21].

Infectious dermatosis is the most common cause of skin infections. Hence, in our study the most common infectious dermatosis was candidiasis (32.2%) and among non infectious dermatosis, pruritic papular eruption (PPE) (20%) accounts highest prevalence.

## Conclusion

This study revealed that high prevalence of mucocutaneous disorder in HIV infected children. Most of the mucocutaneous disorders were secondary to infectious causes and drug related inflammatory condition. Children with advanced immune-suppression are suffering from a wide spectrum of muco-cutaneous disorders. Thus, thorough evaluations of children are recommended in HIV care and treatment centers to address these problems.

## Limitations of the study

The study conducted only descriptive part. Lacking detailed analysis on associated factors is the limitation of the study.

## Additional files

**Additional file 1.** Distribution of specific skin infection as per common skin infection category of children’s living with HIV/AIDS at Hawassa University Comprehensive Specialized Hospital, Hawassa, Ethiopia, 2018.

**Additional file 2.** HIV/AIDS clinical staging and ART related characteristics of the children living with HIV/AIDS in Hawassa Uniersity Comprehensive Specialized Hospital, Hawassa, Ethiopia, 2018 (N = 125).

**Additional file 3.** Association of common skin infections category, Specific CSIs and their determinant factors of the children living with HIV/AIDS in Hawassa University Comprehensive Specialized Hospital, Hawassa, Ethiopia, 2018 (N = 125).

**Additional file 4.** Written informed consent form for the study.
