# Supplementary material for: Patterns of common skin infections among children living with HIV/AIDS in Hawassa City, Ethiopia: a cross sectional study
Source: BMC Res Notes. 2018 Dec 12;11:881. doi: 10.1186/s13104-018-3991-4 (PMC6292031; doi:10.1186/s13104-018-3991-4)
Supplement: Supplementary file 1 — Additional file 1. Distribution of specific skin infection as per common skin infection category of children’s living with HIV/AIDS at Hawassa University Comprehensive Specialized Hospital, Hawassa, Ethiopia, 2018. [file 13104_2018_3991_MOESM1_ESM.docx]

| *Additional File 1:* Distribution of specific skin infection as per common skin infection category of children’s living with HIV/AIDS at Hawassa University Comprehensive Specialized Hospital, Hawassa, Ethiopia, 2018 | | | |
| --- | --- | --- | --- |
| Variable | **Category** | **Number** | **Percentage (%)** |
| Bacterial Skin Infection | Staphylococcal skin infection | 15 | 16.7 |
|  | Bacillary angiomatosis | 3 | 3.3 |
|  | Syphilis | 4 | 4.4 |
| Fungal Skin Infection | Dermatophytosis | 4 | 4.4 |
|  | Candidiasis | 29 | 32.2 |
|  | Cryptococcus’s skin infection | 3 | 3.3 |
|  | TineaCapitis | 3 | 3.3 |
|  | Scalp Impetigo | 4 | 4.4 |
| Viral Skin Infection | Herpes Zoster Skin Infection | 15 | 16.7 |
|  | Herpes Simplex Skin Infection | 18 | 20.0 |
|  | Hairy Leukoplakia | 4 | 4.4 |
|  | MulscumContageosum Skin Infection | 8 | 8.9 |
|  | Verrucae Vulgaris Skin Infection | 1 | 1.1 |
|  | Measles Skin Infection | 2 | 2.2 |
| Arthropod’s related Skin infection | Scabies | 15 | 16.7 |
| Inflammatory related skin Infection | Seborrhea Dermatitis | 9 | 10.0 |
|  | Pruritic Papular Eruption (PPE) | 18 | 20.0 |
|  | Psoriasis and Atopic Eczema | 4 | 4.4 |
|  | Pyomyositis | 2 | 2.2 |
| Cutaneous Malignancy Skin Infection | Kaposi’s Sarcoma | 3 | 3.3 |
| Miscellaneous Skin Infection | Drug Reaction related skin infection such as lipo-dystrophy, nail & hair change | 3 | 3.3 |
