# Supplementary material for: Patterns of common skin infections among children living with HIV/AIDS in Hawassa City, Ethiopia: a cross sectional study
Source: BMC Res Notes. 2018 Dec 12;11:881. doi: 10.1186/s13104-018-3991-4 (PMC6292031; doi:10.1186/s13104-018-3991-4)
Supplement: Supplementary file 2 — Additional file 2. HIV/AIDS clinical staging and ART related characteristics of the children living with HIV/AIDS in Hawassa Uniersity Comprehensive Specialized Hospital, Hawassa, Ethiopia, 2018 (N = 125). [file 13104_2018_3991_MOESM2_ESM.docx]

| **Additional file 2:-** HIV/AIDS clinical staging and ART related characteristics of the children living with HIV/AIDS in Hawassa Uniersity Comprehensive Specialized Hospital, Hawassa, Ethiopia, 2018 (N=125) | | | |
| --- | --- | --- | --- |
| **Variables** | **Categories** | **Number** | **Percent**  **(%)** |
| **Current WHO clinical Stage of the disease** | Stage I | 107 | 85.6 |
|  | Stage II | 15 | 12.0 |
|  | Stage III | 1 | 0.8 |
|  | Stage IV | 2 | 1.6 |
| **Kinds of ART drug taken** | 1^st^ line ART Drug | 70 | 56.0 |
|  | 2^nd^ line ART Drug | 55 | 44.0 |
| **Duration of ART intake** | < 6 month | 11 | 8.8 |
|  | ≥ 6 month | 114 | 91.2 |
| **ART dose Adherence** | Yes | 115 | 92.0 |
|  | No | 10 | 8.0 |
| **ART Schedule Adherence** | Yes | 113 | 90.4 |
|  | No | 12 | 9.6 |
| **Skin related Side effect of ART** | Yes | 3 | 2.4 |
|  | No | 122 | 97.6 |
