# Supplementary material for: Patterns of common skin infections among children living with HIV/AIDS in Hawassa City, Ethiopia: a cross sectional study
Source: BMC Res Notes. 2018 Dec 12;11:881. doi: 10.1186/s13104-018-3991-4 (PMC6292031; doi:10.1186/s13104-018-3991-4)
Supplement: Supplementary file 3 — Additional file 3. Association of common skin infections category, Specific CSIs and their determinant factors of the children living with HIV/AIDS in Hawassa University Comprehensive Specialized Hospital, Hawassa, Ethiopia, 2018 (N = 125). [file 13104_2018_3991_MOESM3_ESM.docx]

| **Additional file 3:** Association of common skin infections category, Specific CSIs and their determinant factors of the children living with HIV/AIDS in Hawassa University Comprehensive Specialized Hospital, Hawassa, Ethiopia, 2018 (N=125) | | |
| --- | --- | --- |
| **Category** | **Number (%)** | **Number (%)** |
|  | **Common skin Infections** | **No Common skin Infections** |
| **ART Dose adherence** |  |  |
| Yes | 80 (64.0) | 35 (28.0) |
| No | 10 (8.0) | 0 (0.0) |
|  | **Bacterial Skin Infection** | **No bacterial Skin Infection** |
| **Current WHO Clinical Stage of HIV** |  |  |
| Stage I | 16 | 91 |
| Stage II | 5 | 10 |
| Stage III | 1 | 0 |
| Stage IV | 0 | 2 |
|  | **Fungal Skin Infection** | **No Fungal Skin Infection** |
| **Duration of ART Intake** |  |  |
| < 6 Months | 7 (5.6) | 4(3.2) |
| ≥ 6 Months | 36 (28.8) | 78(62.4) |
| **Educational Status of caretaker** |  |  |
| Illiterate | 4 | 7 |
| Read and write | 3 | 24 |
| Primary education | 18 | 19 |
| Secondary education | 12 | 17 |
| College/University | 6 | 15 |
|  | **Inflammatory Skin infection** | **No Inflammatory skin infection** |
| ART Dose adherence |  |  |
| Yes | 27(21.6) | 88(70.4) |
| No | 6 (4.8) | 4(3.2) |
| **Immunization Hx of the child** |  |  |
| Fully Immunized | 2(1.6) | 86(68.8) |
| Immunized Some | 0(0.0) | 33(26.4) |
| Not immunized | 1(0.8) | 3(2.4) |
| **Residence of care taker** |  |  |
| Urban | 1 | 113 |
| Rural | 2 | 8 |
|  | **Cutaneous-Malignancy skin infection** | **No Cutaneous Malignancy skin infection** |
| **Current WHO Clinical Stage of HIV** |  |  |
| Stage I | 1 | 106 |
| Stage II | 1 | 14 |
| Stage III | 1 | 0 |
| Stage IV | 0 | 2 |
