# Supplementary material for: Patterns of common skin infections among children living with HIV/AIDS in Hawassa City, Ethiopia: a cross sectional study
Source: BMC Res Notes. 2018 Dec 12;11:881. doi: 10.1186/s13104-018-3991-4 (PMC6292031; doi:10.1186/s13104-018-3991-4)
Supplement: Supplementary file 4 — Additional file 4. Written informed consent form for the study. [file 13104_2018_3991_MOESM4_ESM.pdf]

PARENT/GUARDIAN PERMISSION /INFORMED CONSENT FORM

Parent or Legal Guardian Permission for Child to Participate in a Research Study

[Research Title: Patterns of Common Skin Infections among Children Living with HIV/AIDS in Hawassa City, Ethiopia, cross sectional study]

Patient's information document

Dear participants,

This study is intended to assess the Patterns of Common Skin Infections among Children Living with HIV/AIDS in Hawassa City, Ethiopia, cross sectional study which is expected to forward recommendations to solve the problem. Your child is invited to participate on this project. If you are willing to participate on this project, you need to understand and sign the agreement form. After then you will be interviewed and your child will be examined by dermatologist. You do not need to write your and child's name or to tell your name to the data collector and all your response and the results obtained will be kept confidentially by using coding system. You are being asked to give permission for your child to participate in a research study. Before you give permission for your child to participate, it is important you read the following information and ask as many questions as necessary to be sure you understand what you child is being asked to do.

Investigators

This research project was reviewed and approved by the ethical committee of Hawassa University. If you have any question you can contact any of the following individuals and you may ask any time you want.

Contact address of principal investigator: -<br>Bereket Duko (BSc, MPhi, Assistant Professor)<br>Tell: 0911975900<br>Email: berkole.dad@gmail.com<br>Bedilu Deribe (MSc)<br>Tell: 0933220151<br>Email: bediluderibe2002@gmail.com	Contact address of Co- investigator: -<br>Melese Gebrei (BSc, MSc, Lecturer)<br>Tell: 0911708987<br>Email: natanmeless@gmail.com

Purpose of the Research

This research study is designed to assess the prevalence of common skin problems among children living with HIV/AIDS at Hawassa University Comprehensive Specialized Hospital, Hawassa, Ethiopia, 2017/2018. The study will cross sectional study design. The data from this research will show the scope of the problem in the

study area and information gathered from this study will provide baseline data and serve as an incentive to do larger scale studies in this area that might help to improve the overall care given to children with HIV/AIDS.

Procedures

If you allow your child to participate in this study, you will be asked yours and your child socio-economic characteristics; then dermatologist will assess your child for common skin manifestations. Your child's participation will take approximately 40 minutes. Your child will be asked to assent to participate in this research. He/she can refuse to participate without penalty or can stop participation at any time just by telling the investigator he/she wants to stop. No gift or reward will be given to the child for participation, however: child who will have skin manifestations will be referred to dermatology clinic for further diagnosis and management.

Potential Risks or Discomforts

By participating in this research project, you may fill that it has some discomfort especially on wasting time about 40 minutes. We hope you will participate in the study for the sake of the benefit of the research result. There are no foreseeable physical, psychological, emotional, social or economic risks associated with the study. You have full right to stop participating, either temporarily or permanently.

Potential Benefits of the Research

If you (your child) participate in this research project, there may not be direct benefit to you but your participation more likely help us to meet the research objective. However, if your child has been diagnosed with common skin manifestation, you will have further assessment by dermatologist in dermatology clinic with your permission. Ultimately, this will help us to improve services for children with HIV/AIDS.

Confidentiality and Data Storage

The information collected from this research project will be kept confidential. Information will be stored in a file, without your name, only code number is used. It will not be revealed to anyone except the principal investigator and it will be kept locked with key.

Participation and Withdrawal

You (Your child) have full right to refuse from participating in this research. You (Your child) can choose not to respond to some or all question if you do not want to give your response. If you decide to allow your child to participate, you are free to stop his/her participation without penalty by just stopping and/or telling the investigator. In addition, your child may stop participating by telling the investigator he/she wants to stop.

Questions about the Research

If you have any questions about the research, please ask them now. If you have questions later, you may contact the principal and co-investigators.

This research project has been reviewed and approved by the Institutional Review Board for the Protection of Human Subjects at the Hawassa University.

<u>Parent or Legal Guardian Permission:</u>

I have read the information provided above. I agree to let my child participate in this research study. I also understand my child's assent to participate in this study will be sought. Please return one copy of this consent form and keep one copy for your records.

____

Code of Child (please print)

____ _______________

Signature of Parent/Legal Guardian Date

____ _______________

Signature of Investigator/Data collector Date
